# Discrepancy between medical knowledge and clinical reasoning: evaluating large language models in prenatal diagnosis

**DOI:** 10.3389/fdgth.2026.1917573

**Published:** 2026-09-22

**Authors:** Qiuling Guo, Yanyun Du, Jingcheng Dai, Weiru Lin

**Affiliations:** 1 Department of Ultrasound, Quanzhou Maternal and Child Health Hospital (Quanzhou Children’s Hospital), Quanzhou, China; 2 Department of Gynecology and Obstetrics, Quanzhou Maternal and Child Health Hospital (Quanzhou Children’s Hospital), Quanzhou, China

**Keywords:** artificial intelligence, LLMS, prenatal diagnostic, qualification examination, ultrasound

## Abstract

**Purpose:**

Large language models (LLMs) have demonstrated impressive performance across various medical examinations; however, their competency in subspecialty clinical reasoning, particularly in prenatal diagnosis, remains poorly characterized.

**Materials and methods:**

A cross-sectional comparative study was conducted by using 125 questions from China's nationally standardized Prenatal Ultrasound Screening Qualification Examination. Three LLMs and two human physicians were evaluated. Questions were classified along three independent taxonomic axes: modality, disease system, and question format. Cochran's *Q* test was used for omnibus comparisons, with *post-hoc* pairwise McNemar tests where were significant.

**Results:**

Overall accuracy did not differ significantly among the five test-takers (*p* = 0.126): the chief physician scored highest (89.60%, 112/125), followed by Claude-Sonnet-4.6 (85.60%, 107/125), Grok-4 (84.80%, 106/125), ChatGPT-5 (84.00%, 105/125), and the attending physician (80.00%, 100/125). No significant differences were observed when stratified by modality (text-only, *p* = 0.146; imaging diagnostic questions, *p* = 0.603) or by any individual disease system (all *p* > 0.05). However, performance divergence emerged sharply across question types. In clinical vignette-based questions (*n* = 20), the chief physician (95.00%) significantly outperformed all other test-takers (*p* = 0.016), with a 25% gap over the best-performing LLM (Grok-4, 70.00%). In sequential item sets, Grok-4 (95.74%) and the chief physician (93.62%) both significantly outperformed the attending physician (76.60%; both *p* < 0.05). Conversely, LLMs excelled in knowledge-retrieval tasks, with ChatGPT-5 and Claude-Sonnet-4.6 both achieving 100% accuracy in matching questions. No significant differences were detected among test-takers for factual recall, matching, or multiple-choice multiple-answer questions (all *p* > 0.05).

**Conclusion:**

LLMs are well-positioned to serve as powerful knowledge augmentation tools, but are not yet ready to function as autonomous clinical reasoners in this complex, ethically consequential subspecialty.

## 1 Introduction

Each year, approximately four million neonates worldwide—accounting for 3% of all live births—are affected by congenital anomalies, while millions of affected children are left with lifelong disabilities, imposing a substantial public health and socioeconomic burden (1). Prenatal ultrasonography, as the cornerstone of secondary prevention for birth defects, enables the early detection of lethal and disabling fetal structural malformations, thereby directly improving pregnancy outcomes and reducing the incidence of severe birth defects (2, 3). In China, prenatal screening and diagnosis is classified as a strictly regulated specialized medical technology; practitioners are required to pass provincial-level prenatal screening qualification examinations before obtaining the legally mandated licensure to practice. Accordingly, this examination constitutes the national standard for evaluating the core competencies of prenatal ultrasound specialists in China.

The convergence of artificial intelligence and clinical medicine is advancing at an unprecedented pace, with large language models (LLMs) emerging as one of the most transformative technologies in the field of medical informatics. Kung et al. (4) evaluated the performance of ChatGPT on the United States Medical Licensing Examination in 2023, demonstrating that ChatGPT, without any specialized training or reinforcement, achieved scores at or near the passing threshold across all three examinations, while exhibiting a high degree of concordance and insight in its explanations. Su et al. (5) assessed ChatGPT's performance on 400 questions from Taiwan's 2022 Nursing Licensing Examination in 2024, reporting an overall accuracy of 80.75%; however, substantial variability was observed across different subject areas, with a higher error rate in complex question formats such as clinical scenario-based items, and occasional inconsistencies between the selected answers and their accompanying explanations. With the release of GPT-4V, the first large-scale commercially available large language model capable of directly processing uploaded images for query-based reasoning was officially introduced, thereby shifting scholarly attention from the performance of LLMs on purely text-based examinations to their evaluation in the domain of medical imaging. Huang et al. (6) administered 106 multiple-choice questions from the 2022 American College of Radiology Diagnostic Radiology In-Training Examination to two LLMs—ChatGPT-4o and Gemini Advanced—in 2025, finding that the former achieved a higher overall accuracy (69.8%) than the latter (60.4%), with a more pronounced advantage on image-based questions, whereas the two models performed comparably on text-based items.

Although standardized medical examinations are rigorously designed and well-structured, their assessment emphasis tends to favor factual recall and pattern recognition, while the multidimensional, stepwise, and context-dependent reasoning inherent in real-world clinical decision-making is inadequately captured by such instruments. This discrepancy is particularly pronounced across medical subspecialties, where diagnostic reasoning requires the integration of multimodal data, tolerance of irreducible uncertainty, and the iterative refinement of differential diagnoses amid dynamically evolving clinical conditions. Prenatal diagnosis represents a paradigmatic example of such complex clinical scenarios. Prenatal ultrasound screening occupies a uniquely challenging position within clinical medicine. The discipline demands that practitioners synthesize fetal anatomical and imaging findings, maternal medical history, gestational age, biochemical markers, genetic risk profiles, and population-level epidemiological data to arrive at diagnostic conclusions—conclusions that carry profound implications for pregnancy management and parental counseling (7–9). Unlike most other diagnostic domains, prenatal diagnosis is constrained by the inherent limitations of fetal imaging, including restricted acoustic windows, the dynamic evolution of fetal anatomy across gestational ages, and the inability in most circumstances to directly obtain fetal tissue specimens for testing, thereby compelling clinicians to operate within a persistent state of diagnostic uncertainty (10, 11). Furthermore, the ethical and emotional gravity borne by diagnostic conclusions in this field confers upon clinicians a degree of professional responsibility that far exceeds that of most other medical subspecialties. At present, although the literature on the performance of large language models in the fields of obstetrics and gynecology and prenatal medicine is steadily expanding, the scope of investigation has largely been confined to assessments of general obstetric knowledge or narrow-scope validation of specific imaging interpretation tasks (12, 13). Few studies have employed clinically validated, comprehensive subspecialty examination instruments—tools that encompass the full spectrum of cognitive demands ranging from factual recall to complex clinical reasoning. Concurrently, there is a paucity of research benchmarking the performance of large language models against that of human physicians stratified by varying levels of clinical experience. Moreover, a central question remains insufficiently addressed: whether large language models exhibit differential performance across distinct categories of cognitive tasks within the same clinical domain—a question of critical importance for informing the development of rational clinical deployment strategies.

This study aims to evaluate three of the most representative large language models currently available—namely ChatGPT-5, Claude-Sonnet-4.6, and Grok-4—alongside two clinicians of differing seniority (one chief physician and one attending physician) serving as human comparators, using a standardized prenatal ultrasound screening qualification examination comprising 125 questions. The selection of this examination instrument was guided by specific considerations: first, it encompasses nine major fetal organ systems; second, it incorporates both purely text-based questions and imaging diagnostic questions; and third, it spans five distinct question formats, ranging from factual recall and clinical application to integrative reasoning. Through this multidimensional assessment framework, the present study seeks not only to compare the overall performance of large language models against that of human experts, but more importantly, to delineate the specific cognitive dimensions along which their performance diverges.

## 2 Materials and methods

### 2.1 Study design

This study adopted a cross-sectional comparative design, employing a standardized prenatal ultrasound screening qualification examination to evaluate the diagnostic reasoning capabilities of three representative LLMs and two clinical physicians (Figure 1). The study was conducted between March and April 2026. As this study involved solely the evaluation of artificial intelligence model outputs and the analysis of de-identified examination performance data from human physicians who provided informed consent, ethical concerns were minimal. This study was approved by the Ethics Committee of Quanzhou Maternal and Child Health Hospital.

**Figure 1:**
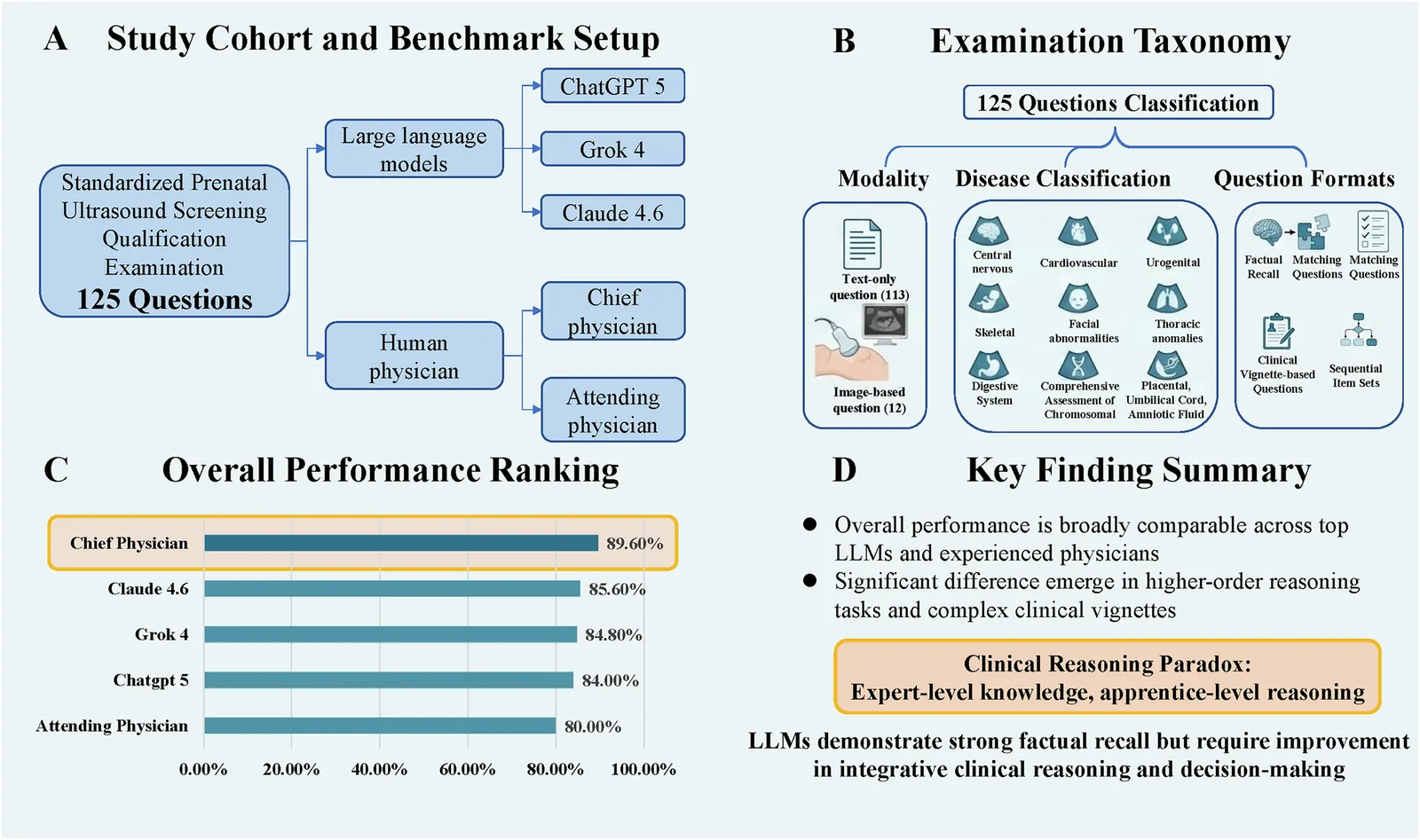
Flowchart and key results of this study.

### 2.2 Exam paper

The examination instrument employed in this study comprised 125 questions derived from China's Standardized Prenatal Ultrasound Screening Qualification Examination. This examination constitutes a mandatory assessment for clinical physicians seeking licensure to practice prenatal ultrasound screening and is widely recognized as a rigorous evaluative tool for both theoretical knowledge and practical diagnostic competence in the field.

The 125 questions were categorized along three independent classification dimensions. First, by question modality, items were divided into text-only questions (*n* = 113) and imaging diagnostic questions (*n* = 12). Imaging diagnostic questions required interpretation in conjunction with prenatal ultrasound images, with image analysis constituting a core component of the diagnostic task. Second, by disease system, questions spanned nine major categories: cardiovascular diseases (*n* = 51), central nervous system diseases (*n* = 7), comprehensive assessment of chromosomal abnormalities (*n* = 13), digestive system diseases (*n* = 10), facial diseases (*n* = 7), placental, umbilical cord and amniotic fluid abnormalities (*n* = 5), skeletal system diseases (*n* = 11), thoracic anomalies (*n* = 6), and urogenital system diseases (*n* = 15). Third, by question format, items were classified into five types in ascending order of cognitive demand: factual recall single-best-answer questions (*n* = 21), clinical vignette-based questions (*n* = 20), sequential item sets (*n* = 47), matching questions (*n* = 16), and multiple-choice multiple-answer questions (*n* = 21). Among these, factual recall single-best-answer questions assess the capacity for direct knowledge retrieval; matching questions require the establishment of associations among related concepts; multiple-choice multiple-answer questions emphasize comprehensive knowledge mastery and discriminative selection ability; clinical vignette-based questions present specific clinical scenarios to evaluate diagnostic integration skills; and sequential item sets consist of multiple interlinked questions that necessitate sustained reasoning informed by dynamically evolving clinical information.

### 2.3 Subject

#### 2.3.1 Clinical physician

Two clinical physicians with differing levels of experience in prenatal ultrasound diagnosis participated in this study. The chief physician possessed over 15 years of subspecialty experience in prenatal ultrasonography, held a senior professional technical title certification, and was engaged in both clinical practice and teaching at a tertiary referral center. The attending physician had 5 to 8 years of clinical experience in obstetric ultrasonography and held an intermediate professional technical title certification. Both physicians completed the examination independently under standardized conditions, without access to any external reference materials during the testing process.

#### 2.3.2 Large language model

Three state-of-the-art large language models were selected for evaluation in this study: ChatGPT-5 (OpenAI Inc., California, United States of America), Claude-Sonnet-4.6 (Anthropic Inc., California, United States of America), and Grok-4 (xAI Inc., California, United States of America). All models were accessed through their respective official web-based interfaces.

A standardized format was employed to present the identical set of 125 questions to each model. For text-only questions, the question stem and answer options were entered directly in textual form; for imaging diagnostic questions, the corresponding ultrasound images were uploaded concurrently with the textual prompt. A unified, standardized system prompt was configured for all models, instructing each to assume the role of a prenatal diagnosis specialist and to select the optimal answer for every question (Supplementary 1). Each question was submitted within an independent conversational session to preclude contextual interference between items.

No prompt engineering techniques—such as chain-of-thought prompting or few-shot example demonstrations—were employed in this study, with the explicit intent of evaluating the baseline performance of each model and simulating the test-taking conditions of a human examinee without specialized prompting. Each model was permitted only a single attempt per question, consistent with the single-response rule governing human examinees.

### 2.4 Statistical analyses

Statistical analyses in this study were performed using SPSS version 26.0 and Origin 2024. Test accuracy was reported as proportions (n/N). Radar plots were constructed to visually depict the performance of each examinee across the various subgroups. Cochran's *Q* test was first employed to assess whether statistically significant differences in accuracy existed among the examinees overall; where significant differences were identified, pairwise comparisons were subsequently conducted using McNemar's test. A *p*-value of <0.05 was considered statistically significant.

## 3 Results

### 3.1 Overall performance of participants

The overall accuracy rates of the five examinees across all 125 questions are detailed in Table 1. The chief physician achieved the highest accuracy at 89.60% (112/125), followed by Claude-Sonnet-4.6 (85.60%, 107/125), Grok-4 (84.80%, 106/125), and ChatGPT-5 (84.00%, 105/125), while the attending physician attained an accuracy of 80.00% (100/125). Although a gradient in performance was observed among the examinees, the overall differences in accuracy across the group did not reach statistical significance (*p* = 0.126).

**Table 1:** Performance of participants categorized by question modality.

Question modality	Attending physician	Chief physician	Chatgpt 5	Grok-4	Claude-Sonnet-4.6	Q value	*p* value
Text-only questions (*n* = 113)	92 (81.42%)	102 (90.27%)	97 (85.84%)	97 (85.84%)	100 (88.50%)	6.180	0.146
Imaging diagnostic questions (*n* = 12)	8 (66.67%)	10 (83.33%)	8 (66.67%))	9 (75.00%)	7 (58.33%)	2.737	0.603
Overall accuracy (*n* = 125)	100 (80.00%)	112 (89.60%)	105 (84.00%)	106 (84.80%)	107 (85.60%)	7.184	0.126

### 3.2 Performance of participants categorized by question modality

The results of stratified analysis by question modality are presented in Table 1. No statistically significant differences in accuracy among the five examinees were observed for either text-only questions (*n* = 113) or imaging diagnostic questions (*n* = 12) (*p* = 0.146 and 0.603, respectively). Among text-only questions, the chief physician maintained the highest accuracy at 90.27%; of the three large language models, Claude-Sonnet-4.6 demonstrated the best performance (88.50%), followed by Grok-4 (85.84%) and ChatGPT-5 (84.96%); the attending physician achieved an accuracy of 81.42%. For imaging diagnostic questions, the chief physician again ranked first (83.33%); among the artificial intelligence models, Grok-4 attained the highest accuracy (75.00%), outperforming both Claude-Sonnet-4.6 and ChatGPT-5 in image interpretation tasks.

### 3.3 Performance of participants categorized by disease system

The accuracy rates of each examinee across questions pertaining to the nine major fetal disease systems are presented in Table 2 and Figure 2A. No statistically significant differences in accuracy among examinees were observed for any disease system (*p* all >0.05), indicating a high degree of comparability in overall performance at the disease system level. Nonetheless, each examinee exhibited distinctive domain-specific proficiency profiles. The chief physician demonstrated the most consistent performance, achieving 100% accuracy in questions related to four disease systems: central nervous system diseases, comprehensive assessment of chromosomal abnormalities, skeletal system diseases, and thoracic anomalies; concurrently, the chief physician attained the highest accuracy among all examinees in questions concerning cardiovascular diseases (86.27%) and urogenital system diseases (93.33%). The three large language models displayed notable yet divergent domain-specific strengths. ChatGPT-5 achieved 100% accuracy in questions related to central nervous system diseases, comprehensive assessment of chromosomal abnormalities, and placental, umbilical cord and amniotic fluid abnormalities; Grok-4 attained 100% accuracy in central nervous system disease questions; and Claude-Sonnet-4.6 achieved perfect scores in questions pertaining to facial diseases, placental, umbilical cord and amniotic fluid abnormalities, and skeletal system diseases.

**Table 2:** Performance of participants categorized by disease system.

Disease system	Attending physician	Chief physician	Chatgpt 5	Grok-4	Claude-Sonnet-4.6	*Q* value	*p* value
Cardiovascular diseases (*n* = 51)	37 (72.55%)	44 (86.27%)	43 (84.31%)	43 (84.31%)	43 (84.31%)	6.809	0.146
Central nervous system diseases (*n* = 7)	6 (85.71%)	7 (100.00%)	7 (100.00%)	7 (100.00%)	6 (85.71%)	3.000	0.558
Comprehensive assessment of chromosomal abnormalities (*n* = 13)	11 (64.82%)	13 (100.00%)	13 (100.00%)	12 (92.31%)	12 (92.31%)	4.000	0.406
Digestive system diseases (*n* = 10)	8 (80.00%)	7 (70.00%)	6 (60.00%)	6 (60.00%)	7 (70.00%)	2.800	0.592
Facial diseases (*n* = 7)	6 (85.71%)	6 (85.71%)	6 (85.71%)	6 (85.71%)	7 (100.00%)	4.000	0.406
Placental, umbilical cord and amniotic fluid abnormalities (*n* = 5)	4 (80.00%)	4 (80.00%)	5 (100.00%)	4 (80.00%)	5 (100.00%)	2.400	0.663
Skeletal system diseases (*n* = 11)	10 (90.91%)	11 (100.00%)	9 (81.81%)	10 (90.91%)	11 (100.00%)	4.000	0.406
Thoracic anomalies (*n* = 6)	5 (83.33%)	6 (100.00%)	4 (66.67%)	5 (83.33%)	4 (66.67%)	3.500	0.478
Urogenital system diseases (*n* = 15)	13 (86.67%)	14 (93.33%)	12 (80.00%)	13 (86.67%)	12 (80.00%)	2.154	0.707

**Figure 2:**
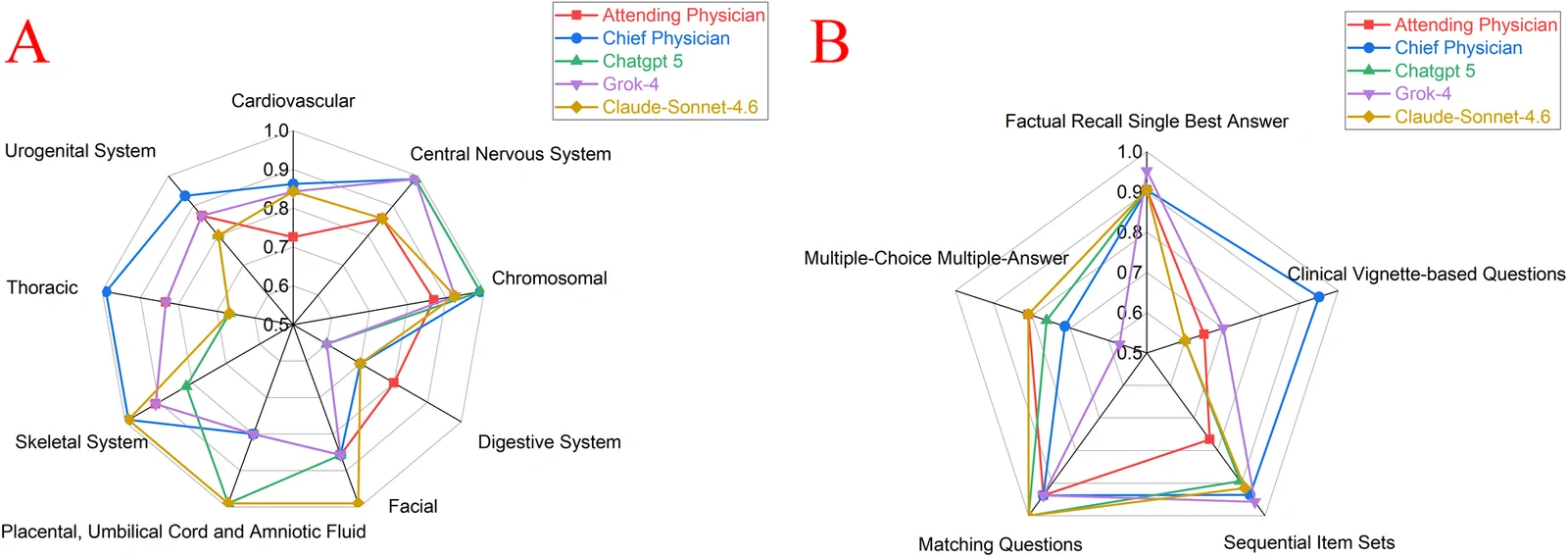
Radar charts of each participant's performance by disease system **(A)** and question type **(B)**.

### 3.4 Performance of participants categorized by question type

The analysis stratified by question format yielded the most diagnostically informative findings of this study (Table 3, Figure 2B). Among the five question formats evaluated, statistically significant differences in accuracy across examinees were identified for clinical vignette-based questions (*n* = 20) and sequential item sets (*n* = 47) (*p* = 0.016 and <0.05, respectively), whereas no statistically significant differences were observed for factual recall single-best-answer questions, matching questions, or multiple-choice multiple-answer questions (*p* all >0.05).

**Table 3:** Performance of participants categorized by question type.

Question type	Attending physician	Chief physician	Chatgpt 5	Grok-4	Claude-Sonnet-4.6	*Q* value	*p* value
Factual recall single best answer (*n* = 21)	19 (90.48%)	19 (90.48%)	19 (90.48%)	20 (95.24%)	19 (90.48%)	0.889	0.926
Clinical vignette-based questions (*n* = 20)	13 (65.00%)	19 (95.00%)	12 (60.00%)	14 (70.00%)	12 (60.00%)	12.143	0.016
Sequential item sets (*n* = 47)	36 (76.60%)	44 (93.62%)	42 (89.36%)	45 (95.74%)	43 (91.49%)	15.152	0.004
Matching questions (*n* = 16)	15 (93.75%)	15 (93.75%)	16 (100.00%)	15 (93.75%)	16 (100.00%)	2.000	0.736
Multiple-choice multiple-answer (*n* = 21)	17 (80.95%)	15 (71.43%)	16 (76.19%)	12 (57.14%)	17 (80.95%)	6.370	0.173

For clinical vignette-based questions, the chief physician achieved an accuracy of 95.00%, and McNemar's test revealed that this accuracy was significantly higher than that of all other examinees (*p* = 0.016). Among the three LLMs, Grok-4 demonstrated the relatively best performance with an accuracy of 70.00%, while ChatGPT-5 and Claude-Sonnet-4.6 achieved even lower accuracy rates in this question format. For sequential item sets, Grok-4 ranked first with an accuracy of 95.74%, marginally surpassing the chief physician (93.62%). McNemar's test indicated that the accuracy rates of both the chief physician and Grok-4 were significantly higher than that of the attending physician (76.60%, *p* < 0.05 for both comparisons), while all remaining pairwise comparisons yielded no statistically significant differences (*p* all >0.05). In stark contrast, the large language models excelled in question formats centered on knowledge retrieval. Both ChatGPT-5 and Claude-Sonnet-4.6 achieved 100% accuracy in matching questions. For factual recall single-best-answer questions and multiple-choice multiple-answer questions, the performance of all five examinees was closely comparable, with no single examinee demonstrating a clear dominant advantage.

## 4 Discussion

The findings of this study reveal that large language models achieved overall accuracy rates comparable to or approaching expert-level performance on a comprehensive prenatal diagnosis examination, matching or even surpassing the performance of a mid-career clinical physician. However, on tasks requiring context-dependent clinical reasoning and the integration of multidimensional information to arrive at a diagnosis, a systematic and significant decline in model performance was observed.

In factual recall single-best-answer questions and matching questions—formats that emphasize knowledge retrieval and associative pattern matching—LLMs performed at or above the level of both human physicians, with ChatGPT-5 and Claude-Sonnet-4.6 achieving perfect scores on matching questions. This may be attributable to the fact that Transformer-based language models encode vast repositories of medical knowledge within their model parameters and are capable of retrieving this information with remarkably high accuracy (14).

However, in clinical vignette-based questions—items that demand the integration of patient history, clinical context, gestational age information, and various diagnostic findings to formulate a coherent clinical judgment—even the best-performing LLM exhibited an accuracy gap of up to 25 percentage points relative to the chief physician. This pronounced disparity, observed within the same examination and the same clinical domain, provides compelling evidence that the cognitive architecture underpinning LLM performance is fundamentally distinct from the cognitive processes of experienced human physicians. Clinical vignette-based questions in prenatal diagnosis are not merely advanced versions of factual recall single-best-answer questions; rather, they constitute a qualitatively different category of cognitive task. Answering such questions requires the examinee to construct a mental model of the clinical scenario, identify diagnostically salient information within the question stem while distinguishing it from distractors, probabilistically weight various pieces of information in light of the specific clinical context, and ultimately formulate a diagnostic hypothesis that accounts for the entirety of the available evidence. By contrast, the mechanism by which LLMs process clinical cases operates through sequential token-by-token prediction. Although this mechanism is capable of generating text that mimics the process of clinical reasoning, it is inherently unable to construct and manipulate internal representations of clinical scenarios in the manner of a human expert. When an experienced prenatal diagnosis specialist reads a clinical case, a complete illness script is activated, integrating anatomical knowledge, principles of embryological development, associated chromosomal abnormalities, and gestational age-specific considerations into a coherent diagnostic framework. While LLMs encompass all of these individual knowledge elements within their training corpora, they struggle to achieve the contextual integration required to transform disparate facts into clinical judgments.

Within the broader trend of LLMs generally underperforming on reasoning-intensive tasks, a noteworthy exception merits attention: Grok-4 achieved the highest accuracy among all examinees on sequential item sets at 95.74%, marginally surpassing even the chief physician's 93.62%. Sequential item sets are designed as a series of interrelated questions centered on a single clinical scenario, where each subsequent question introduces additional clinical information and requires the examinee to refine or extend their diagnostic reasoning accordingly. Although such items likewise demand sustained contextual engagement, they provide a structured reasoning framework and confine the reasoning task to specific decision nodes. This structured framework aligns closely with the conditional text generation capabilities of LLMs, as each successive question effectively serves as a new prompt built upon the cumulative clinical context. In contrast, clinical vignette-based questions present all relevant and irrelevant information simultaneously, requiring the examinee to independently identify, prioritize, and integrate diagnostically meaningful elements—constituting a less structured and cognitively more demanding task. The markedly divergent performance of LLMs across these two ostensibly reasoning-intensive question formats suggests that the underlying issue is not merely one of question difficulty, but rather reflects a specific deficit in the models' capacity for unstructured clinical integration—namely, the inability to autonomously construct a diagnostic framework from complex, unfiltered clinical narratives. This distinction carries important implications for guiding the clinical deployment of LLMs: when provided with structured, stepwise clinical information, these models may serve as effective reasoning support tools; however, when tasked with autonomously processing unstructured and complex diagnostic scenarios, they are liable to produce unreliable outputs.

The inclusion of two human physicians at different experience levels as comparators in this study provides an indispensable reference framework for interpreting the performance of LLMs. The chief physician consistently maintained a leading position, with a particularly pronounced advantage on reasoning-intensive tasks—a finding that corroborates the classical paradigm of medical expertise development, whereby accumulated clinical experience transforms fragmented factual knowledge into an integrated, context-sensitive clinical cognitive framework (15). The attending physician achieved an overall accuracy of 80.00%, which, although a commendable result, was lower than that of both the chief physician and all three LLMs. This finding carries dual implications. On one hand, it demonstrates that the medical knowledge repositories of current state-of-the-art LLMs have surpassed those of mid-career physicians who have completed formal training and are actively practicing in clinical settings. This represents a remarkable achievement and underscores the substantial potential of LLMs as educational and knowledge support tools, particularly for physicians in the early stages of their careers or for those practicing in primary care facilities where access to specialist consultation resources is limited (16). On the other hand, while LLMs surpassed the attending physician in overall accuracy, they remained inferior to the chief physician in clinical reasoning tasks—a phenomenon that reveals a central conclusion: current LLMs occupy a paradoxical position within the hierarchy of medical professional competence, possessing specialist-level knowledge reserves yet demonstrating only apprentice-level clinical reasoning and judgment capabilities. This characterization of “knowledge giants, clinical apprentices” precisely encapsulates the essence of the clinical reasoning paradox and simultaneously provides direct guiding principles for the integration of such tools into clinical workflows. Another noteworthy finding is that the attending physician outperformed all other examinees, including the chief physician, on questions related to digestive system diseases. Although this result did not achieve statistical significance, it is consistent with perspectives in the literature on the “expertise reversal effect,” suggesting that the relationship between experience and performance is not a simple positive correlation across all subspecialty domains. Possible explanations for this phenomenon include recent completion of targeted training on specific diseases by the attending physician, relative overconfidence on the part of the chief physician in diagnosing conditions with low prevalence, or random fluctuations inherent to a small question sample. Regardless of the specific underlying cause, this finding serves as a reminder that professional competence is domain-specific, and one cannot assume that either human physicians or LLMs will possess uniformly balanced and excellent capabilities across all areas within a given specialty.

It is noteworthy that, for imaging diagnostic questions, although the chief physician ranked first with an accuracy of 83.33%, no statistically significant differences were observed between LLMs and human physicians. This result is somewhat unexpected, given that prenatal ultrasound image interpretation is generally regarded as a perceptual skill uniquely mastered by human practitioners. This may be attributable to the fact that, while the imaging diagnostic questions in this study incorporated ultrasound images, they were presented in a multiple-choice format, thereby constraining the diagnostic task to selecting from predetermined options rather than rendering an original diagnosis based on an unconstrained visual field. This question design enabled LLMs to leverage their cross-modal visual-language capabilities to identify key diagnostic features within the images and match them to the most plausible answer option, even though the models' holistic perceptual comprehension of the images may not be as thorough as that of human sonographers (17). Furthermore, state-of-the-art multimodal LLMs have been exposed to vast quantities of medical imaging data during their training process, which establishes a foundation for performing structured image interpretation tasks, even though these models do not possess visual perceptual capabilities in the true sense (18). It must be emphasized that prenatal ultrasound examination in clinical practice encompasses the entire process of dynamic, real-time imaging: the physician must actively manipulate the transducer to optimize image display, mentally reconstruct the three-dimensional fetal anatomy from two-dimensional cross-sectional planes, and correlate ultrasonographic findings with real-time fetal movements and hemodynamic data. These core elements of prenatal ultrasonography are entirely unrepresented in static multiple-choice question formats and remain a domain that current LLMs are unable to engage with. Accordingly, the results from the imaging diagnostic questions in this study reflect only model performance on constrained image recognition tasks and should not be construed as evidence that LLMs possess clinical ultrasound diagnostic capabilities.

Several limitations of this study warrant acknowledgment. First, the human comparator group comprised only two physicians, which constrains the generalizability of the human performance benchmark. Considerable individual variation exists among physicians at the same experience level, and the performance of these two physicians may not be fully representative of their respective career stages. Future studies should incorporate a larger physician sample drawn from multiple institutions to establish a more robust reference standard. Second, although the examination instrument employed in this study consisted of nationally standardized questions with comprehensive coverage, it represents only a single assessment at a single point in time; LLM performance may vary with different examination instruments, question banks, or testing periods. Third, each question was submitted to the models only once, without prompt engineering optimization. While this approach maximized the ecological validity of the study by simulating an unassisted clinical consultation scenario, it may have underestimated the potential performance of the models under optimized prompting strategies such as chain-of-thought reasoning, self-consistency sampling, or retrieval-augmented generation. Fourth, the publicly available general-purpose LLMs evaluated in this study are language-oriented systems rather than dedicated medical image-analysis models, despite their multimodal image-input capabilities and the direct upload of ultrasound images to the three web-based LLM interfaces. Therefore, the results from the (12) static ultrasound image questions should be interpreted cautiously and should not be generalized to specialized medical image interpretation. Future studies and clinical applications should employ or develop dedicated AI systems trained and validated for prenatal ultrasound analysis, potentially integrating them with LLMs for clinical reasoning and decision support. Fifth, other frontier LLMs, including Gemini, were not evaluated, which may have limited the breadth of model comparisons. Future studies should include Gemini, whose multimodal architecture may demonstrate distinct performance in imaging diagnosis and clinical reasoning, helping clarify whether the observed discrepancy between knowledge retrieval and clinical reasoning reflects a general characteristic of frontier LLMs or a model-specific phenomenon.

## 5 Conclusion

In the domains of theoretical knowledge and static image assessment pertaining to prenatal screening and diagnosis, LLMs have demonstrated capabilities that match or marginally surpass those of senior chief physicians. Nevertheless, the integrative reasoning capacity of human experts when confronted with complex, multivariable clinical cases retain a definitive advantage. The future of medical artificial intelligence lies not in replacing sonographers, but in empowering those physicians who master and judiciously utilize AI tools, thereby redefining the boundaries of efficiency and precision in prenatal diagnosis.

## Data Availability

The raw data supporting the conclusions of this article will be made available by the authors, without undue reservation.
