# Supplementary material for: Discrepancy between medical knowledge and clinical reasoning: evaluating large language models in prenatal diagnosis
Source: Front Digit Health. 2026 Sep 22;8:1917573. doi: 10.3389/fdgth.2026.1917573 (PMC13640009; doi:10.3389/fdgth.2026.1917573)
Supplement: Supplementary file 1 [file Supplementaryfile1.docx]

I. Type A1 Questions

Prompt Version 1 (General Non-Image-Based Questions)

Act as a professional in the field of prenatal ultrasound screening and complete the following Type A1 single-best-answer questions. Requirements are as follows: (1) Question Type Description: Each question has only one optimal correct answer, and the remaining options are distractors; (2) Answer Requirements: Directly list the question number + correct answer option for each question.

The questions are as follows:

Prompt Version 2 (Image-Based Diagnostic Questions)

Based on your professional knowledge of prenatal ultrasound diagnosis, complete the following Type A1 image-based single-best-answer questions. Requirements are as follows: (1) Question Type Description: As shown in the attachments, each question is accompanied by a corresponding prenatal ultrasound image, and there is only one optimal correct answer per question; (2) Answer Requirements: First, determine the fetal anatomical structure or abnormal sign corresponding to the image based on the ultrasound image description provided; then, combine the question options to provide the question number + correct answer option.

The image descriptions + questions are as follows:

II. Type A2 Questions (Case-Based Single-Best-Answer Questions + Partial Image-Based Questions)

Prompt Version 1 (General Case-Based Questions)

From the perspective of a clinical prenatal ultrasound physician, complete the following Type A2 case-based single-best-answer questions. Requirements are as follows: (1) Question Type Description: Each question provides a clinical case summary of a pregnant woman (including gestational age, medical history, ultrasound findings, etc.), with only one optimal correct answer per question; (2) Answer Requirements: First, sort out the key information in the case (e.g., gestational age, fetal biometric parameters, ultrasound abnormal manifestations); then, based on the principles of prenatal ultrasound diagnosis, provide the question number + correct answer option.

The case summaries + questions are as follows:

Prompt Version 2 (Case + Image-Based Questions)

Combine prenatal ultrasound case information and imaging features to complete the following Type A2 case-based image-based single-best-answer questions. Requirements are as follows: (1) Question Type Description: Each question includes a pregnant woman’s case summary + textual description of ultrasound images, with only one optimal correct answer per question; (2) Answer Requirements: Synthesize the case information (gestational age, medical history, etc.) and ultrasound image features to clarify the ultrasound diagnostic conclusion of the fetus; then match the conclusion with the question options.

The case summaries + questions are as follows:

III. Type A3 Questions (Case-Based Grouped Questions + Partial Image-Based Questions)

Based on the clinical diagnosis and treatment logic of prenatal ultrasound, complete the following Type A3 case-based grouped questions. Requirements are as follows: (1) Question Type Description: This question type shares one core set of case/ultrasound image data, with multiple interrelated multiple-choice questions attached, each having only one optimal correct answer; (2) Answer Requirements: First, conduct a comprehensive analysis of the core information in the shared case/images (e.g., basic conditions of the pregnant woman, key fetal ultrasound signs); (3) List the correct answer option for each question in the order of question numbers.

The shared case/image descriptions + grouped questions are as follows:

IV. Type B Questions (Matching Questions)

Complete the following Type B matching questions for prenatal ultrasound screening. Requirements are as follows: (1) Question Type Description: This question type includes one set of alternative answers and multiple question stems. Each question stem can only select one most matching option from the alternative answers, and the alternative answers can be used once, multiple times, or not used at all; (2) Answer Requirements: List the corresponding correct answer option for each question stem in the order of question stem numbers.

The alternative answers + question stems are as follows:

V. Type X Questions (Multiple-Choice Questions with Multiple Correct Answers)

Based on your professional knowledge of prenatal ultrasound screening, complete the following Type X multiple-choice questions with multiple correct answers. Requirements are as follows: (1) Question Type Description: Each question has two or more correct answers; no points will be awarded for selecting more, fewer, or incorrect options; (2) Answer Requirements: Directly list the question number + all correct answer options for each question.

The questions are as follows:
